# Identification of cancer-related miRNA-lncRNA biomarkers using a basic miRNA-lncRNA network

**DOI:** 10.1371/journal.pone.0196681

**Published:** 2018-05-01

**Authors:** Guangle Zhang, Cong Pian, Zhi Chen, Jin Zhang, Mingmin Xu, Liangyun Zhang, Yuanyuan Chen

**Affiliations:** 1 Department of Mathematics, College of Science, Nanjing Agricultural University, Nanjing, Jiangsu, China; 2 Ministry of Agriculture Key Lab of Molecular Biology of Crop Pathogens and Insects, Institute of Insect Science, Zhejiang University, Hangzhou, Zhejiang, China; Universitat de Barcelona, SPAIN

## Abstract

LncRNAs are regulatory noncoding RNAs that play crucial roles in many biological processes. The dysregulation of lncRNA is thought to be involved in many complex diseases; lncRNAs are often the targets of miRNAs in the indirect regulation of gene expression. Numerous studies have indicated that miRNA-lncRNA interactions are closely related to the occurrence and development of cancers. Thus, it is important to develop an effective method for the identification of cancer-related miRNA-lncRNA interactions. In this study, we compiled 155653 experimentally validated and predicted miRNA-lncRNA associations, which we defined as basic interactions. We next constructed an individual-specific miRNA-lncRNA network (ISMLN) for each cancer sample and a basic miRNA-lncRNA network (BMLN) for each type of cancer by examining the expression profiles of miRNAs and lncRNAs in the TCGA (The Cancer Genome Atlas) database. We then selected potential miRNA-lncRNA biomarkers based on the BLMN. Using this method, we identified cancer-related miRNA-lncRNA biomarkers and modules specific to a certain cancer. This method of profiling will contribute to the diagnosis and treatment of cancers at the level of gene regulatory networks.

## Introduction

Non-coding RNAs (ncRNAs) are a class of RNA molecules that lack protein-coding potential. Several important classes of ncRNA are transfer RNAs (tRNAs), ribosomal RNAs (rRNAs), microRNAs (miRNAs), small nucleolar RNAs (snoRNA), small nuclear RNAs (snRNA), and long non-coding RNAs (lncRNAs). LncRNAs are defined as ncRNAs of at least 200 nucleotides (nt) in length that molecularly resemble mRNA [1, 2]. Mounting evidence indicates that lncRNAs play important roles in regulating gene expression at the transcriptional, post-transcriptional and epigenetic levels [3–5]. Dysregulated lncRNAs have been implicated in many complex diseases, such as cardiovascular disease, breast cancer, and pancreatic cancer [6–12]. MiRNAs are ncRNAs 20–24 nucleotides in length that regulate gene expression by binding to the 3’ untranslated region (3’UTR) of target mRNAs to inhibit target mRNA translation and silence target expression [13, 14]. A large number of studies have suggested that miRNAs participate in many important biological processes and that the aberrant expression of miRNAs might cause diseases such as breast cancer and lung cancer [15–18].

A biomarker is generally any substance that defines the state of a particular disease or biological function of an organism. Biomarkers are often used to examine normal biological processes, pathogenic progressions, or pharmacologic responses to a therapeutic intervention. The identification of effective disease-related biomarkers is important for the prevention and diagnosis of diseases. Furthermore, biomarkers contribute to the research and development of drugs for the treatment of a given disease. Thus, it is important to identify disease-related biomarkers. Recent studies have focused on the identification of signal-molecule signatures [19–22]. However, complex diseases usually result from the dysregulation of regulatory networks rather than the dysfunction of a single molecule. Furthermore, lncRNAs comprise a large proportion of the transcriptome. Many recent studies have shown that lncRNAs, along with miRNAs, can act as competing endogenous RNAs (ceRNAs) to regulate target mRNAs, thereby playing an important role in the initiation and progression of cancer. Moreover, lncRNAs can act as miRNA targets to sequester miRNA away from other endogenous targets. Recently, attempts have been made to identify miRNA-lncRNA biomarkers [8, 23, 24]. For instance, Wang et al. [8] identified the functional lncRNA-associated competing triplets (lncACTs) from expression data. They also constructed a regulatory network to identify competing modules and calculated the risk scores for good prognosis using the identified biomarkers. However, the functional mechanisms underlying these miRNA-lncRNA associations remain unclear. Thus, it is important to develop an effective method by which to study the function of miRNA-lncRNA interactions and to identify novel miRNA-lncRNA biomarkers of cancers by analyzing the miRNA-lncRNA interaction network.

As our understanding of diseases mechanisms and RNA function improves, an increasing number of studies have focused on biological regulatory networks to identify novel disease-related biomarkers. In 2015, Wang et al. identified functional RNA pairs by computing the Pearson correlation coefficients and p-values based on expression profiles [8]. Zhang et al. developed a competing network using miRcode, StarBase and miRTarBase in HCC, with a focus on the hub nodes of the network [23]. In 2017, Guo et al. assessed the joint predictive power of miRNAs and lncRNAs using miRNA and lncRNA expression data, and identified an integrated miRNA-lncRNA signature of OV patients with wild-type copies of BRCA1/2 [24]. Nevertheless, these methods have limitations. The samples must be accompanied by historical information, and individual-specific networks are not constructed. In addition, some methods are unable to discover miRNA-lncRNA interactions with significant functional roles. With the rise of cancer morbidity and the advent of personalized medicine, now is the time to develop a method for constructing individual miRNA-lncRNA regulatory networks that are patient-specific and can identify effective cancer-related miRNA-lncRNA biomarkers. At present, no approach exists to solve this problem. Here, we construct basic miRNA-lncRNA networks (BMLNs) for individual cancers, and an individual specific miRNA-lncRNA network (ISMLN) for each disease sample by applying statistical methods. We identify cancer-related miRNA-lncRNA biomarkers by analyzing the BMLNs and ISMLNs. Our results indicate that these methods can be used to identify crucial biomarkers ignored by differential expression analysis. The identified miRNA-lncRNA biomarkers are characteristic of cancer-specific functional modules and can be used to distinguish individual cancer samples.

## Materials and methods

### Data collection

A total of 12727 lncRNA and 1046 miRNA RNA-seq normalized expression values were downloaded from The Cancer Genome Atlas (TCGA) data portal (https://cancergenome.nih.gov/). TCGA is a public and comprehensive genomics data repository that is used to document and identify cancer-associated molecules. We selected six types of cancer for study: breast invasive carcinoma (BRCA), kidney renal clear cell carcinoma (KIRC), lung adenocarcinoma (LUAD), lung squamous cell carcinoma (LUSC), prostate adenocarcinoma (PRAD), and thyroid carcinoma (THCA). We compared the samples of miRNAs with the samples of lncRNAs. If the samples have the expression profiles of miRNAs and lncRNAs simultaneously, we retain them for further analysis. The number of tumor samples and non-tumor samples remaining after preprocessing are shown in Table 1 and the main clinicopathological characteristics of 6 cancer cohorts are shown in S1 Table.

**Table 1:** The number of cancer samples remaining after preprocessing.

Cancer abbreviation	Cancer type	Number of non-tumor samples	Number of tumor samples
**BRCA**	Breast invasive carcinoma	84	508
**KIRC**	Kidney renal clear cell carcinoma	67	185
**LUAD**	Lung adenocarcinoma	19	420
**LUSC**	Lung squamous cell carcinoma	9	67
**PRAD**	Prostate adenocarcinoma	52	370
**THCA**	Thyroid carcinoma	59	496

The number of non-tumor samples and tumor samples represents the quantity of samples that have expression values of miRNAs and lncRNAs simultaneously.

In addition, we collected experimentally validated and predicted miRNA-lncRNA associations from the starBase v2.0 [25], NPInter v3.0 [26], and miRcode [27] databases. It is reasonable to use the experimentally supported miRNA-lncRNA interactions. However, the validated pairs are limited. We integrated the predicted interactions into the study. After unifying the names of miRNAs and lncRNAs, we finally obtained 155653 lncRNA-miRNA associations for further analysis by the uniting of miRNA-lncRNA pairs after removing redundancy. Here, the names of miRNAs and lncRNAs are unified based on miRBase 22 release and LNCipedia 4.0, respectively.

### Constructing an ISMLN from a single sample

**Fig 1:**
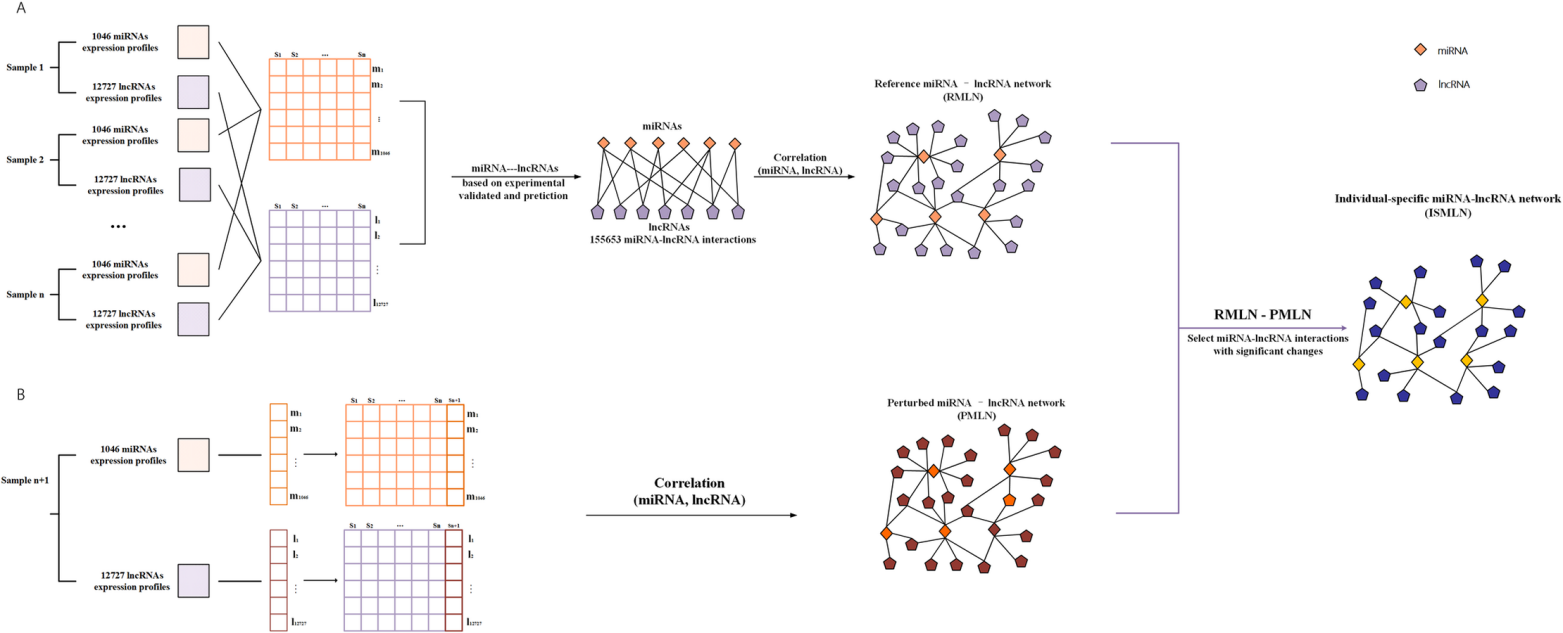
Flowchart detailing the construction of an individual-specific miRNA-lncRNA network (ISMLN). (A) Construction of the reference miRNA-lncRNA network. After selecting n non-tumor samples as the reference samples and 155653 miRNA-lncRNA associations as the basic edges, the reference network is constructed by computing the Pearson Correlation Coefficients (PCCs) based on miRNA and lncRNA expression data. (B) After the addition of the n+1th sample, which is a tumor sample, to the reference network, a perturbed miRNA-lncRNA network is built by calculating the PCCs. The difference between the above two miRNA-lncRNA networks is regarded as the individual-specific miRNA-lncRNA network (ISMLN).

Based on the group of available control samples, the ISMLN of a single sample or individual was constructed by the statistical perturbation method. Fig 1 details the workflow utilized in this study. We included 155653 lncRNA-miRNA associations as the basic dataset to identify candidate cancer-related biomarkers as well as edge biomarkers from the candidate biomarkers. Let S = {*s*_1_,*s*_2_,⋯,*s*_*n*_} represent the n samples with similar or common characters in the control group, and let $m_{i}=(m_{i}^{1},m_{i}^{2},\cdots ,m_{i}^{n})$ and $l_{j}=(l_{j}^{1},l_{j}^{2},\cdots ,l_{j}^{n})$ represent the expression profiles of miRNA *i* and lncRNA *j*, respectively, in the n control samples. We constructed a reference regulatory network by calculating the Pearson Correlation Coefficients (PCCs) of 155653 miRNA-lncRNA pairs based on the expression data (Fig 1A). The reference regulatory network is a background network that remains stable even when the number of reference samples is changed. To obtain the individual specific miRNA-lncRNA network (ISMLN), we added a single tumor sample *S*_*n*+1_ to the reference network; that is, the perturbed miRNA-lncRNA regulatory network was constructed by calculating the PCCs between $m_{i}=(m_{i}^{1},m_{i}^{2},\cdots ,m_{i}^{n},m_{i}^{n+1})$ and $l_{j}=(l_{j}^{1},l_{j}^{2},\cdots ,l_{j}^{n},l_{j}^{n+1})$ (Fig 1B). Next, obtained a differential regulatory network by determining difference between the reference network and perturbed network. If the expression of sample *S*_*n*+1_ differs from the samples in the control group, significant changes will emerge in the regulatory network after the addition of sample *S*_*n*+1_; this differential network can be used to characterize the sample *S*_*n*+1_. Otherwise, the changes between reference network and perturbed network will be insignificant (i.e., the sample *S*_*n*+1_ should be a member of control group). To better characterize the sample-specificity of sample *S*_*n*+1_, we retained only the edges of the perturbed network with statistically significant differences (i.e., we searched for edges with a significant differential PCC (Δ*PCC*)) (Fig 1B). Finally, the network of cancer-related biomarkers was built by utilizing the miRNA-lncRNA pairs with a significant Δ*PCC*.

### Constructing a BMLN for each type of cancer

**Fig 2:**
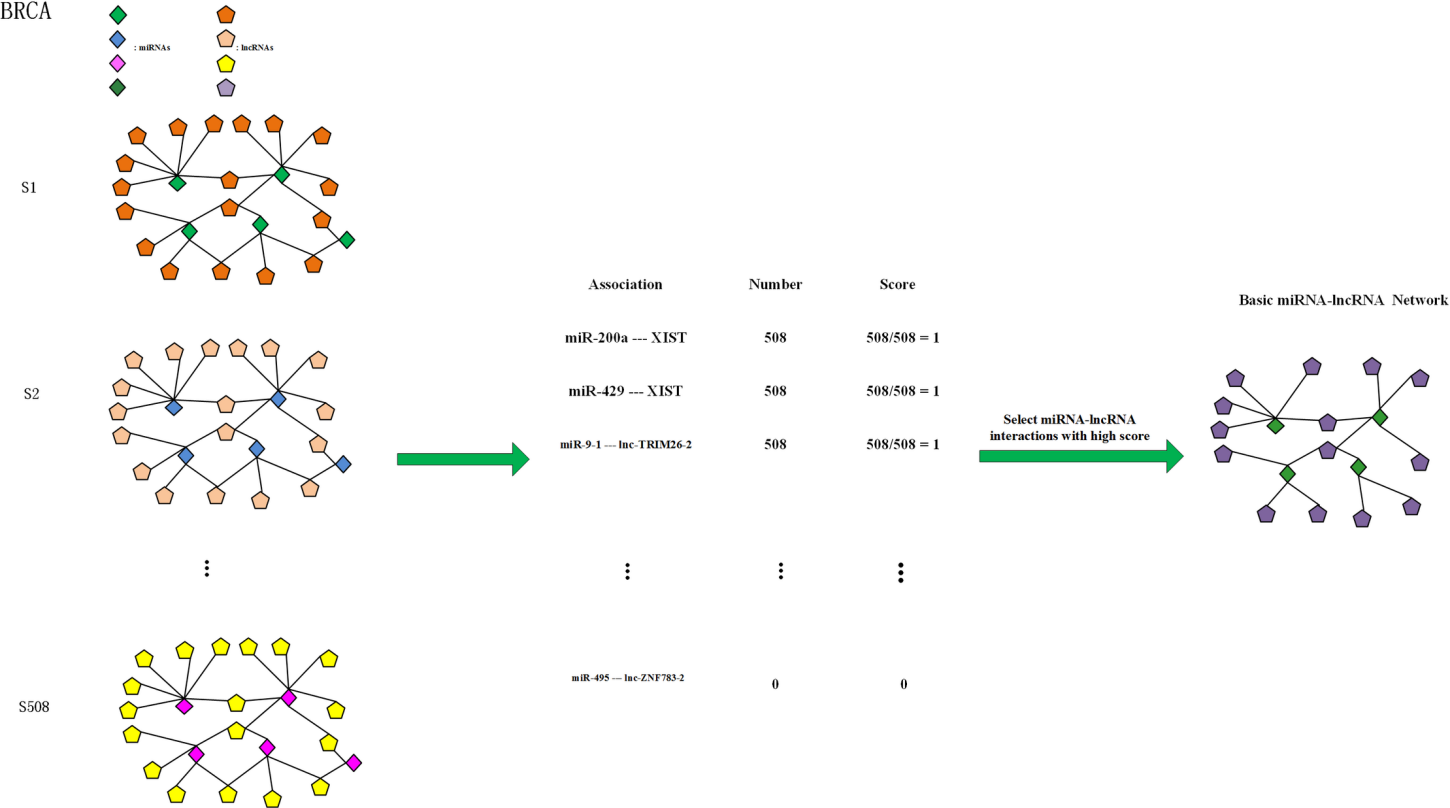
The BMLN for each type of cancer. Taking BRCA as an example, we obtained 508 ISMLNs. We then counted the number of samples with significant changes for each miRNA-lncRNA pair and calculated the significance score. For example, the miR-200a-XIST interaction was significantly altered in all 508 samples, so the corresponding significance score was 508/508 = 1. Similarly, the significance scores of all 155653 miRNA-lncRNA pairs were calculated. We then ranked the miRNA-lncRNA pairs in descending order based on their significance scores. Finally, we chose only the miRNA-lncRNA interactions with high scores for further analysis.

The individual-specific miRNA-lncRNA networks were constructed based on the aforementioned method. The significant miRNA-lncRNA pairs were selected by Z-test statistical analysis. The cutoff value for significant Δ*PCC*s is confirmed by identifying the value that makes the p-value of the Δ*PCC* matrix smaller than and as close as possible to 0.05. We next counted the number of samples whose Δ*PCC* changed significantly for each miRNA-lncRNA pair. As shown in Fig 2, only the miRNA-lncRNA edges with higher scores were retained in the final network. Thus, we obtain a basic miRNA-lncRNA network (BMLN) for each type of cancer. Using BRCA as an example, after obtaining the individual specific miRNA-lncRNA regulatory networks, we counted the number of samples that exhibit a significant change for each miRNA-lncRNA pair. For example, the miR-200a-XIST pair is significantly altered in all 508 BRCA samples, so the significance score is 1 (508/508). The formula for the significance score is as follows:

$$Sigificance\;Score({miRNA}_{i},{lncRNA}_{j})=N_{1}/N$$ (1)

where N_1_ represents the number of cancer samples exhibiting a significant change for a given miRNA_i_-lncRNA_j_ pair, and N is the total number of cancer samples.

Similarly, we calculated the scores of all 155653 miRNA-lncRNA associations and ranked the relations in descending order based on the scores. In the next analysis, we focused on the relations with higher scores, which we refer to as candidate miRNA-lncRNA biomarkers.

### Selecting the potential miRNA-lncRNA edge biomarkers

Using similar steps for paired non-tumor samples, we obtained the Δ*PCC* matrix of all non-tumor samples. Taking the Δ*PCC* values of candidate miRNA-lncRNA biomarkers as classification features, we applied the Random Forests (RF) algorithm in MATLAB R2013a to recognize the cancer samples and paired non-tumor samples, and identified the important features of candidate miRNA-lncRNA biomarkers using the RF classifier. We then ranked the candidate miRNA-lncRNA biomarkers in descending order based on the features contribution, and selected the relations with significance scores greater than 0.8 as potential miRNA-lncRNA edge biomarkers.

To determine the accuracy of this classification model, 10-fold cross-validation was conducted to distinguish the cancer samples from the non-tumor samples based on the potential edge biomarkers uncovered by the RF algorithm. To evaluate the performance of the selected features, we drew Receiver Operating Characteristic (ROC) curves and calculated the Area Under Curves (AUC) for distinct features. The AUC value ranged from [0,1]. The larger the AUC, the better the performance of the classification model. When the AUC = 0.5, this model is random.

### Calculating the Activity Scores of miRNAs involved in candidate biomarker miRNA-lncRNA interactions

**Fig 3:**
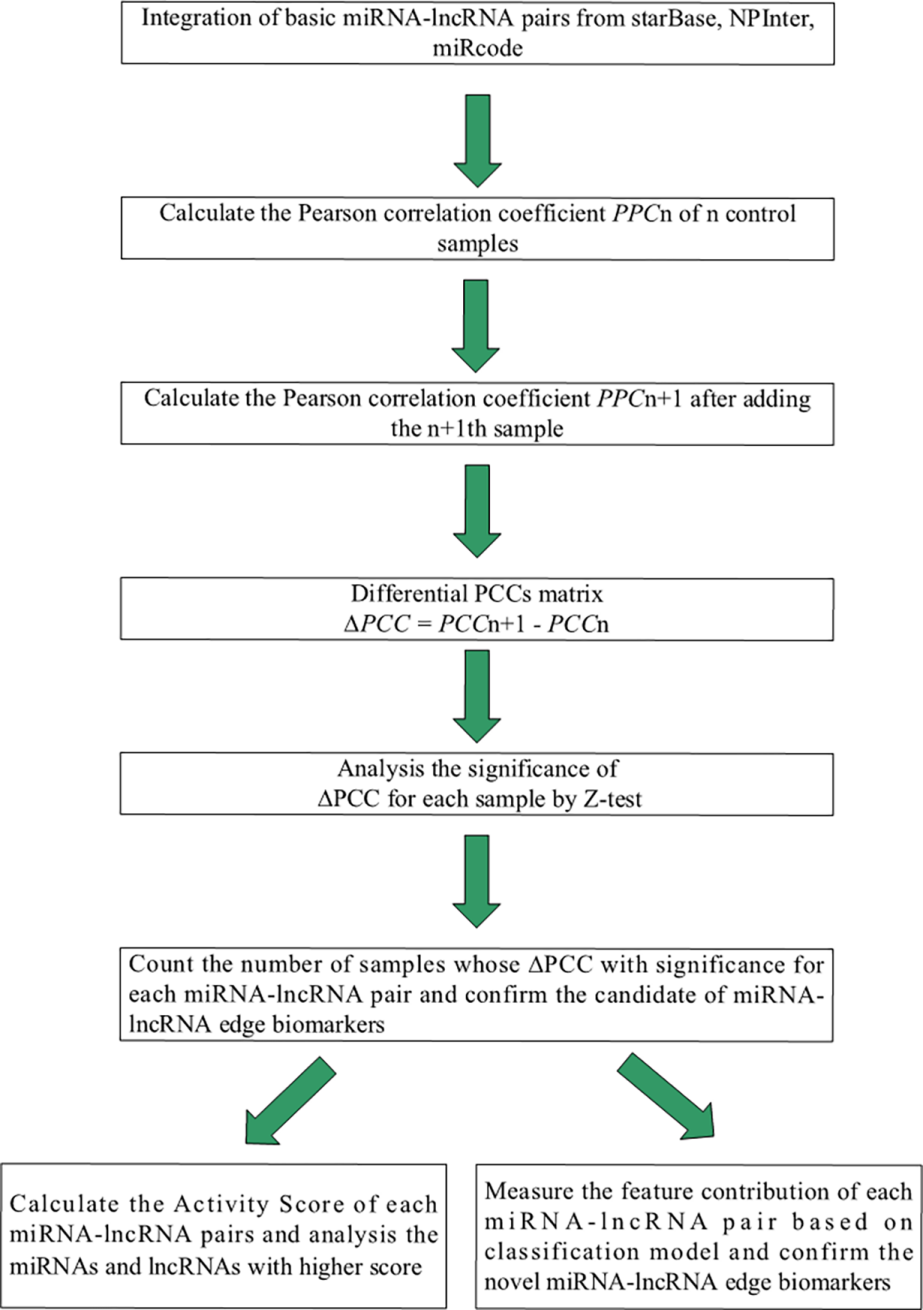
Entire flowchart.

Because the number of RNA molecules involved in the 155653 miRNA-lncRNA interactions is inhomogeneous, we propose the Activity Scores to further analyze the miRNAs involved in the candidate miRNA-lncRNA pairs. The Activity Score of a miRNA is calculated by the following formula:

$$Activity\;Score({miRNA}_{i})=\sum_{j=0}^{k}S({miRNA}_{i},{lncRNA}_{j})\times \left(\frac{C_{1}}{C_{2}}\right)$$ (2)

where S(miRNA_i_, lncRNA_j_) represents the significance score of the miRNA_i_-lncRNA_j_ pair, k is the number of candidate miRNA-lncRNA pairs, C_1_ represents the frequency of miRNA_i_ among the candidate miRNA-lncRNA pairs, and C_2_ is the frequency of the miRNA_i_ among all 155653 miRNA-lncRNA associations. Fig 3 diagrams this procedure.

## Results

### Breast invasive carcinoma (BRCA)

**Fig 4:**
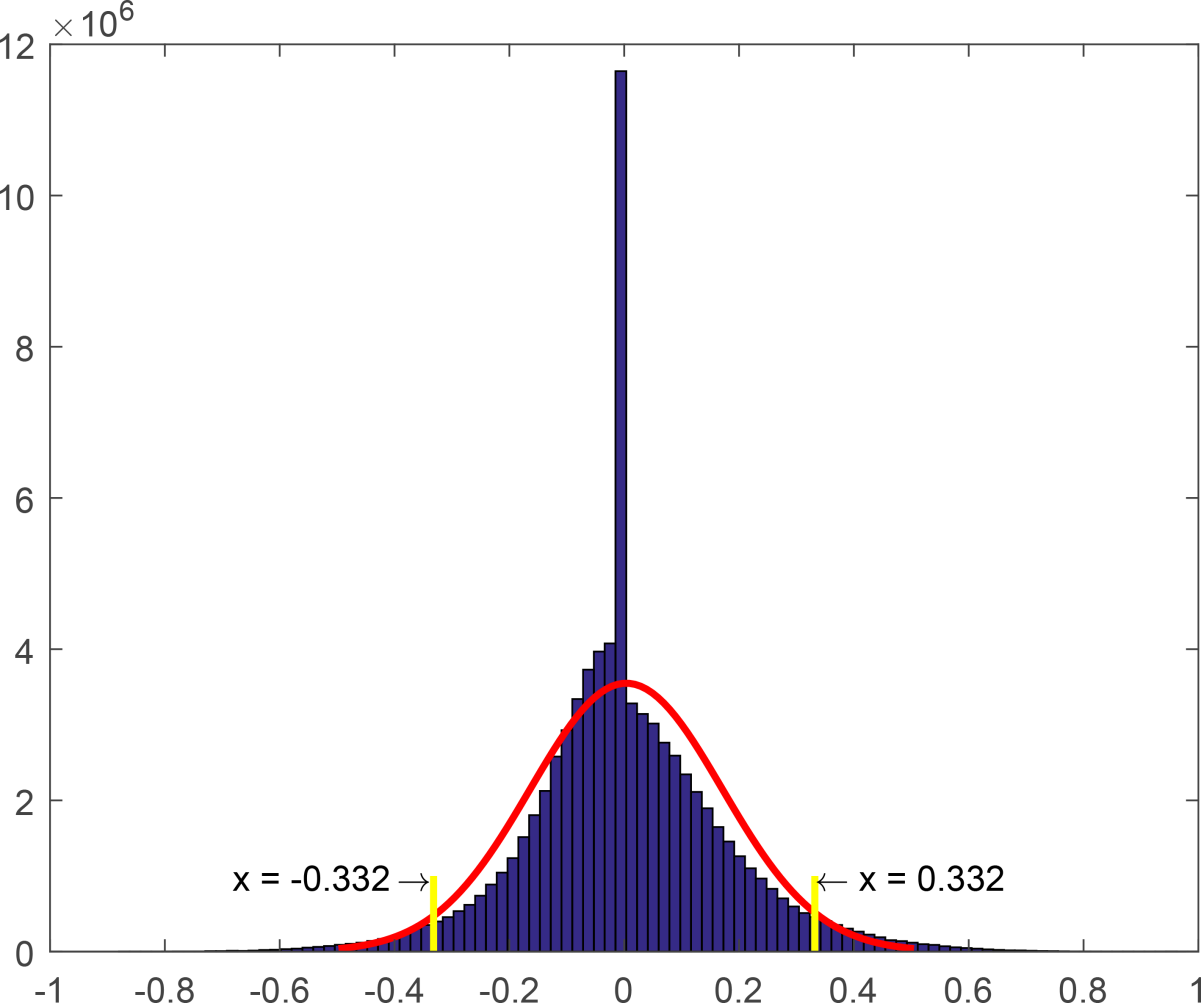
The distribution of ΔPCC values. The cutoff of significant ΔPCC are X = 0.332 and X = -0.332, respectively. The red curve is the normal distribution curve of ΔPCC.

Breast cancer is the most frequent cancer in females. Recently, the rising incidence and mortality of breast cancer are serious health threats to women. Unfortunately, the mechanism of breast cancer remains unclear [28–30]. Here, we focus on the selection of miRNA-lncRNA biomarkers of breast cancer using a novel method proposed in this study. First, we constructed a reference regulatory background network of 155653 miRNA-lncRNA associations using the expression profiles of 84 adjacent non-tumor samples. Second, we successively added a single tumor sample to the background network to generate a perturbed regulatory network and to obtain a differential PCC matrix. Third, we computed a significance score for each miRNA-lncRNA pair based on the significance cutoff value (Fig 4). Fourth, the 8508 miRNA-lncRNA pairs with significance scores greater than 0.8 were selected as candidate edge biomarkers (S2 Table). To further determine the relationship between these candidate biomarkers and breast cancer, the Δ*PCC* values of the candidate edge biomarkers were used to sort breast tumor samples from adjacent non-tumor samples. After, the features importance scores of these relations were obtained. Finally, we obtained a list of candidate biomarkers with higher features importance scores, which are potential miRNA-lncRNA biomarkers for breast cancer.

### The reliability of potential edge biomarkers

The causes of complex diseases can nearly always be traced to the dysregulation of molecular interactions rather than a single molecule. Many network-based methods have been proposed to reliably predict disease-related biomarkers for distinguishing samples with distinct phenotypes. However, these network-based approaches present common limitations. These methods are always developed based on previously known information about diseases. Moreover, when we diagnose the status of a new sample, these network-based methods often fail at identifying characteristics of the new sample. In contrast, the approach proposed herein can be used to construct an ISMLN for each sample, even with little known information. ISMLN provides a novel method for exploring disease-related interactions among molecules, thereby contributing to the development of personalized medicine.

**Fig 5:**
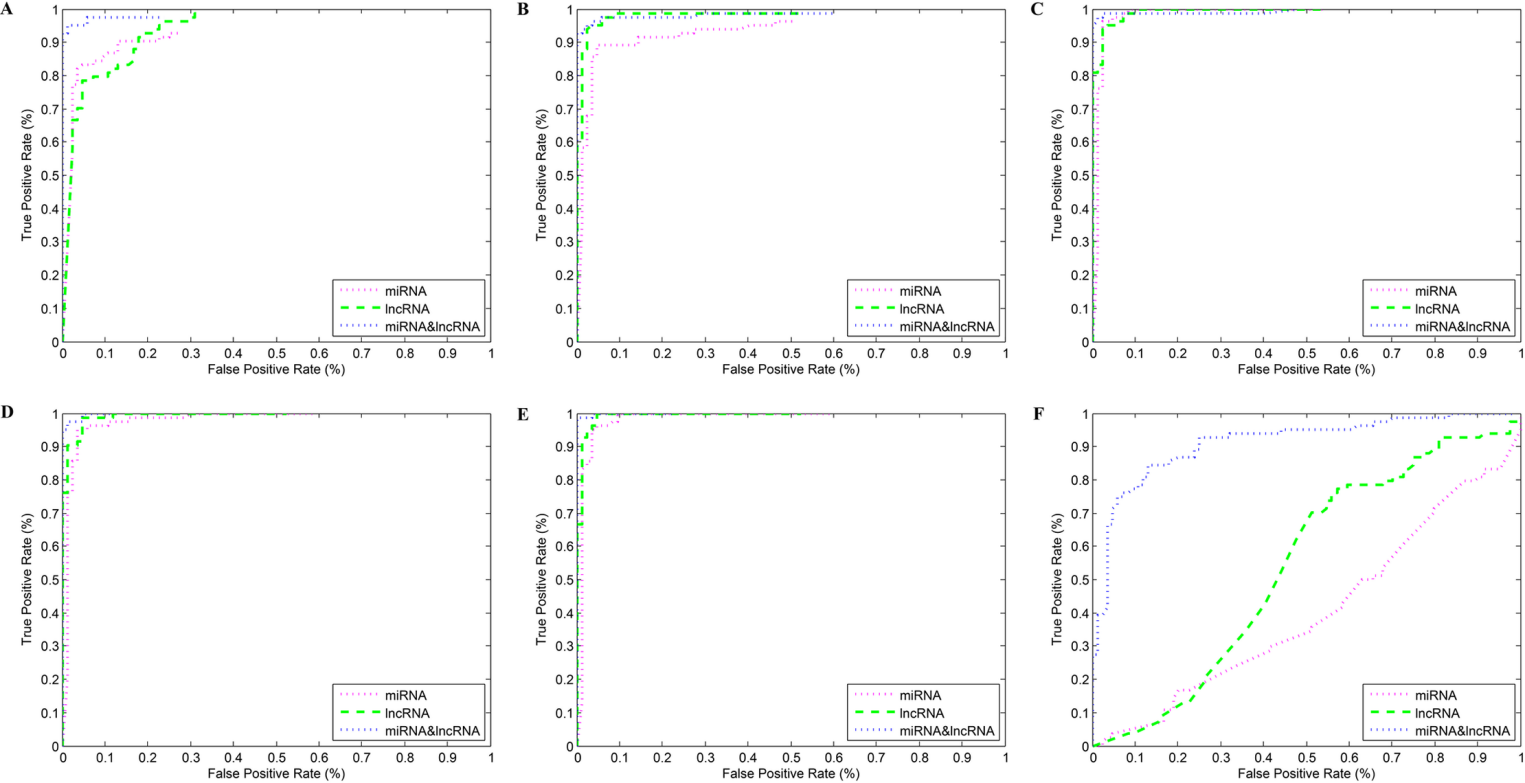
ROC of distinct features. A-F show the ROC curves of classification results that take the top 1, 2, 3, 4, or 5 and last 5 potential miRNA-lncRNA edge biomarkers and the differentially expressed RNAs as the features, respectively.

To determine whether the potential biomarkers are effective, we applied the Δ*PCC* values of the potential edge biomarkers as features to identify samples with distinct phenotypes. Since the performance of classification models is different, we selected three machine learning algorithms (support vector machine (SVM), random forest (RF) and Voting based extreme learning machine (V-ELM)) to classify the samples with different phenotypes based on the top 5 miRNA-lncRNA associations in BMLN. These three machine learning algorithms are classical classification algorithms and have their own advantages. Here, we constructed three different classifiers using three algorithms based on the same dataset. The AUC can reflect the performance of different classifiers. Overall, the AUC of SVM algorithm was 0.9897, the AUC of RF algorithm was 0.9994 and the AUC of V-ELM was 0.9678. It was clear that the RF algorithm outperformed the other two algorithms, so we selected the RF algorithm in this study. Here, an RF algorithm with 10-fold cross-validation was used to classify the breast tumor samples and adjacent non-tumor samples. Moreover, we selected the top 1, 2, 3, 4, or 5 and last 5 potential miRNA-lncRNA edge biomarkers as well as the differentially expressed miRNAs and lncRNAs as features to classify the BRCA samples and adjacent non-tumor samples, respectively. As shown in Fig 5 and Table 2, we found that the mean AUC of the node biomarkers for the top differentially expressed miRNAs and lncRNAs were 0.9626 and 0.9809, respectively. However, the mean AUC of edge biomarkers for the top five potential biomarkers was 0.9923. The maximum AUC of node biomarkers was 0.9942, but for edge biomarkers, we were able to achieve a maximum AUC of 0.9996. Clearly, both node biomarkers and edge biomarkers perform well for identifying the phenotypes of distinct samples. To further assess the performance of node biomarkers and edge biomarkers, node biomarkers among the bottom five differentially expressed RNA molecules and edge biomarkers among the bottom five potential edge biomarkers were chosen to classify the BRCA tumor samples and adjacent non-tumor samples for the same training datasets and testing datasets. We found that the AUC of the last 5 edge biomarkers achieved a mean AUC as high as 0.9126, yet for node biomarkers, the mean AUC was 0.47105 and the maximum AUC was 0.5405. Thus, it is clear that the edge biomarkers selected in this study are more robust than the node biomarkers for classifying the phenotypes of distinct samples. In other words, the edge miRNA-lncRNA biomarkers chosen by us are reliable and more effective.

**Table 2:** The AUC of distinct features of node biomarkers and edge biomarkers.

	Top1	Top2	Top3	Top4	Top5	Last5
**Node** **(miRNA)**	0.9272	0.9381	0.9853	0.9792	0.9832	0.4016
**Node** **(lncRNA)**	0.9410	0.9833	0.9931	0.9931	0.9942	0.5405
**Edge** **(miRNA-lncRNA)**	0.9836	0.9855	0.9943	0.9986	0.9996	0.9126

### Differential expression analysis of RNA molecules in potential biomarkers

**Fig 6:**
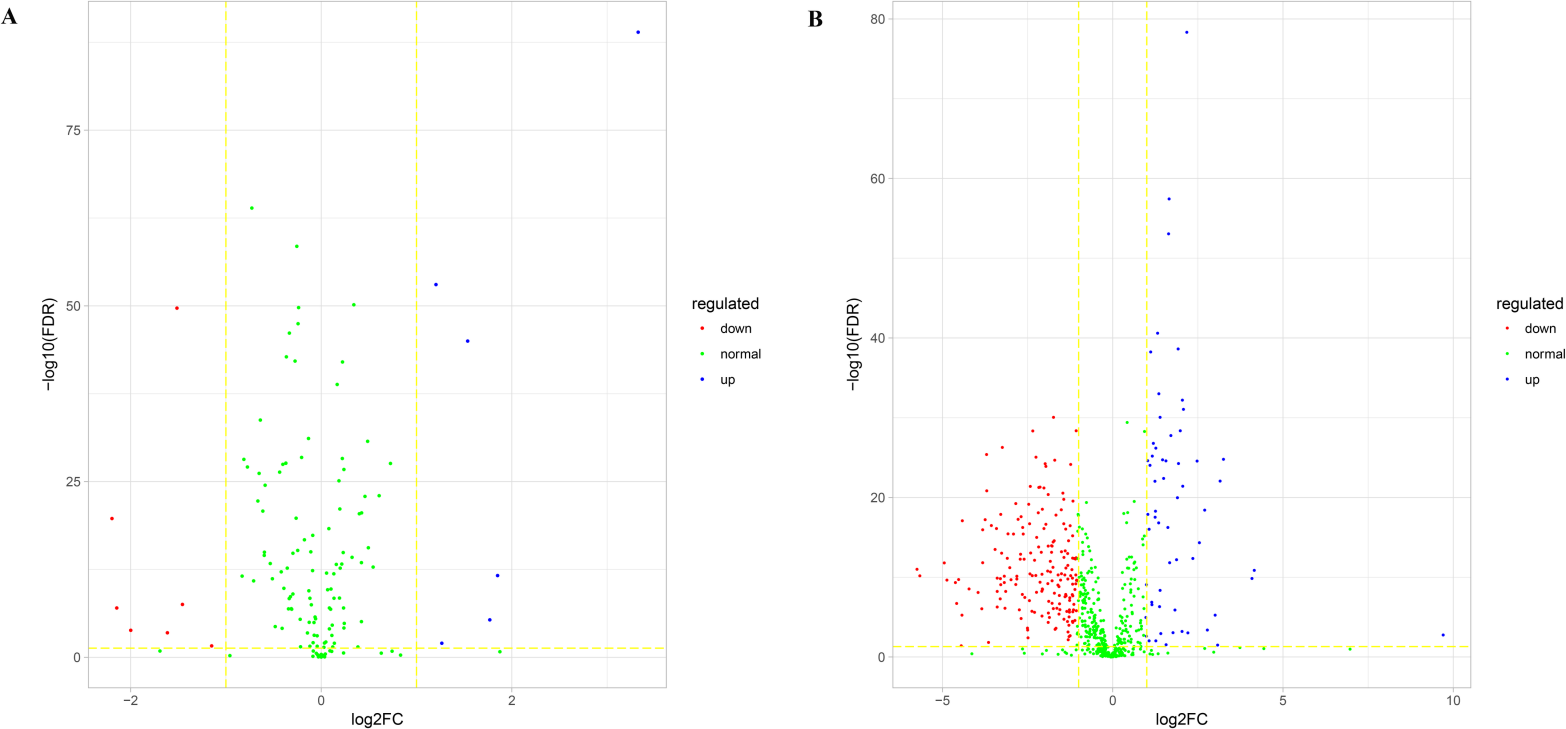
Volcano plots of potential miRNA and lncRNA edge biomarkers. (A) Volcano plot of potential miRNA edge biomarkers. (B) Volcano plot of potential lncRNA edge biomarkers. Green nodes represent RNAs that are not differentially expressed, red nodes represent RNAs that are downregulated, and blue nodes represent RNAs that are upregulated.

Performing differential expression analysis for RNA molecules is one common pretreatment for exploiting disease-related biomarkers [31–34]. Yao et al developed a computational method to identify novel mRNA, miRNA and lncRNA markers of diabetic pancreatic cancer (PaC). At first they identified differentially expressed genes, miRNAs and lncRNAs between diabetic PaC and non-diabetic PaC patients using edgeR package. They further examined their correlations by utilizing clinical information and selected the prognostic RNAs using KM curves finally [31]. Zhang et al identified distinctly expressed miRNAs, lncRNAs and mRNAs, and lncRNAs acting ceRNA based on the miRNA-lncRNA-mRNA interaction network using GO and pathway analyses for differentially expressed mRNAs [32]. The expression profiles of these differentially expressed molecules are then used to calculate PCCs among the RNA molecules, which are in turn used to construct an RNA regulatory network. However, this procedure might lead to the loss of important information. Here, differential expression analysis was applied in the 138 miRNAs and 600 lncRNAs included among the 1025 potential miRNA-lncRNA edge biomarkers. Fig 6 shows volcano plots of the miRNAs and lncRNAs comprising the potential edge biomarkers. We identified merely 13 differentially expressed (DE) miRNAs among 138 miRNAs (Fig 6A), and 6 miRNA among the 13 miRNAs was upregulated. Fig 6B shows the volcano plot of 660 lncRNAs, of which 241 are differentially expressed. Interestingly, only 59 of the lncRNAs were upregulated; almost all DE lncRNAs were downregulated. Furthermore, the top 5 miRNA-lncRNA edge biomarkers include 2 miRNAs and 5 lncRNAs, 3 lncRNAs are differentially expressed. Thus, it is possible to obtain effective edge biomarkers that are ignored by differential expression analysis.

Based on these results, we have learned that it is possible for RNAs that are not differentially expressed which are, by definition, not detected in analyses of differential expression to be important disease-related biomarkers. For example, mir-200a is a key regulator of cancer metastasis, and the miR-200 family is known to promote EMT [35, 36]. MiR-429 inhibits the migration and invasion of breast cancer cells, and induces apoptosis by targeting XIAP in triple-negative breast cancer [37, 38]. Some studies have indicated that XIST participates in the progression of breast cancer [39–41]. TUG1 is known to promote cell proliferation in breast cancer [42–44]. If differential expression analysis is performed to identify candidate biomarkers, these important regulators would be overlooked. In contrast, our method allows the construction of an individual-specific miRNA-lncRNA regulatory network for each sample, which can then be used to identify important biomarkers that are ignored by other methods. In summary, our method permits the identification of biological “dark matter” that might be important for disease diagnosis and therapy.

### The Activity Scores of potential biomarkers miRNAs

Although an increasing number of studies have focused on the roles of lncRNAs, relatively little is known about their biological function. In contrast, we know much more about miRNAs. For example, Li et al. [45, 46] identified associations between miRNAs and diseases, and constructed the comprehensive HMDD database for public use. This database was recently updated to HMDD v2.0. Jiang et al. [47] constructed a comprehensive database (miR2Disease) of dysregulated miRNAs in various human disease contexts. These databases provide a point of entry to query whether a miRNA functions in a particular disease or exhibits a relevant expression pattern.

**Fig 7:**
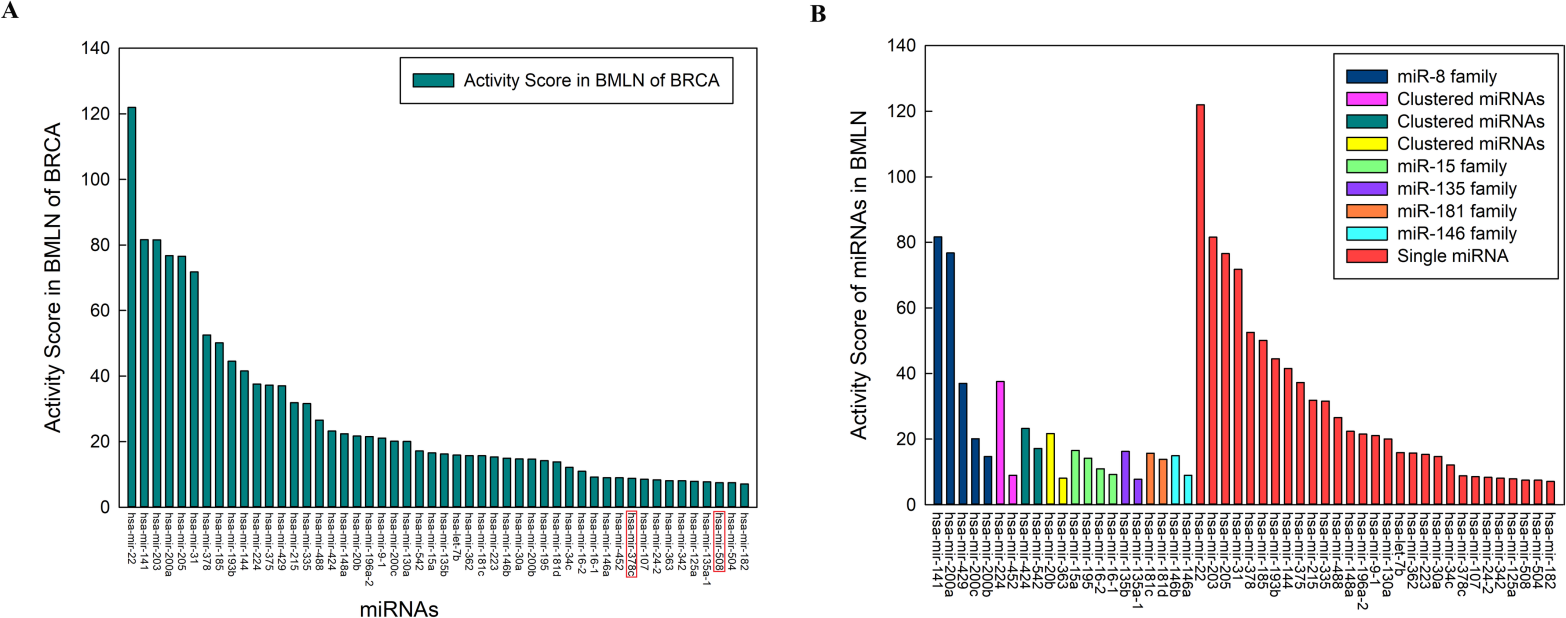
Activity Scores of candidate edge biomarker miRNAs. (A) The top 50 Activity Scores of miRNAs participating in potential miRNA-lncRNA biomarkers in BMLN of BRCA. (B) The categories of top 50 miRNAs based on the Activity Score in BMLN of BRCA.

If a miRNA related to a lncRNA is closely related to a certain disease, it follows that the lncRNA must also be related to this disease. To characterize the performance of potential edge biomarkers, we exploited preexisting miRNA data to depict the functions of predicted lncRNA biomarkers. We therefore defined the Activity Scores of miRNAs to measure the associations between miRNAs and breast cancer and to exploit the functions of lncRNAs and the associations between lncRNAs and cancers. The top 50 Activity Scores of 240 miRNAs in candidate edge biomarker miRNAs are shown in Fig 7A (The Activity Scores of 240 miRNAs are shown in S3 Table). Based on information gathered from the HMDD v2.0 and miR2Disease databases, only 2 of these 50 miRNAs are not related to breast cancer in any study. The Activity Scores of these 2 miRNAs are very small, ranking them near the bottom among miRNAs. These results illustrate that the miRNAs identified using our method are reliable and that the lncRNAs involved in the corresponding miRNA-lncRNA pairs are likely to perform well. Furthermore, the miRNAs ranking among the top 50 in terms of Activity Score (Fig 7B) can be divided into three categories: members of a miRNA family, members of a miRNA cluster, and signal miRNAs. As illustrated in Fig 7B, there are 29 single miRNAs with high Activity Scores. However, large-scale studies have reported they involvement in the progression of breast cancer [48–55]. The miRNA with the highest Activity Score is miR-22. Many researchers have reported that the miR-22 is associated with breast cancer and plays important roles in the development of the disease [56, 57]. MiR-224 and miR-452 belong to the same miRNA cluster and have been implicated in the development of breast cancer in various ways [58–61]. Feng et al. [58] reported that miR-224 down-regulates the expression of fizzled 5 to inhibit the proliferation and migration of breast cancer cells. MiR-452, as a tumor-inhibitor of breast cancer, targets the RAB11A gene directly [60].

### Biomarkers of multiple cancers

**Fig 8:**
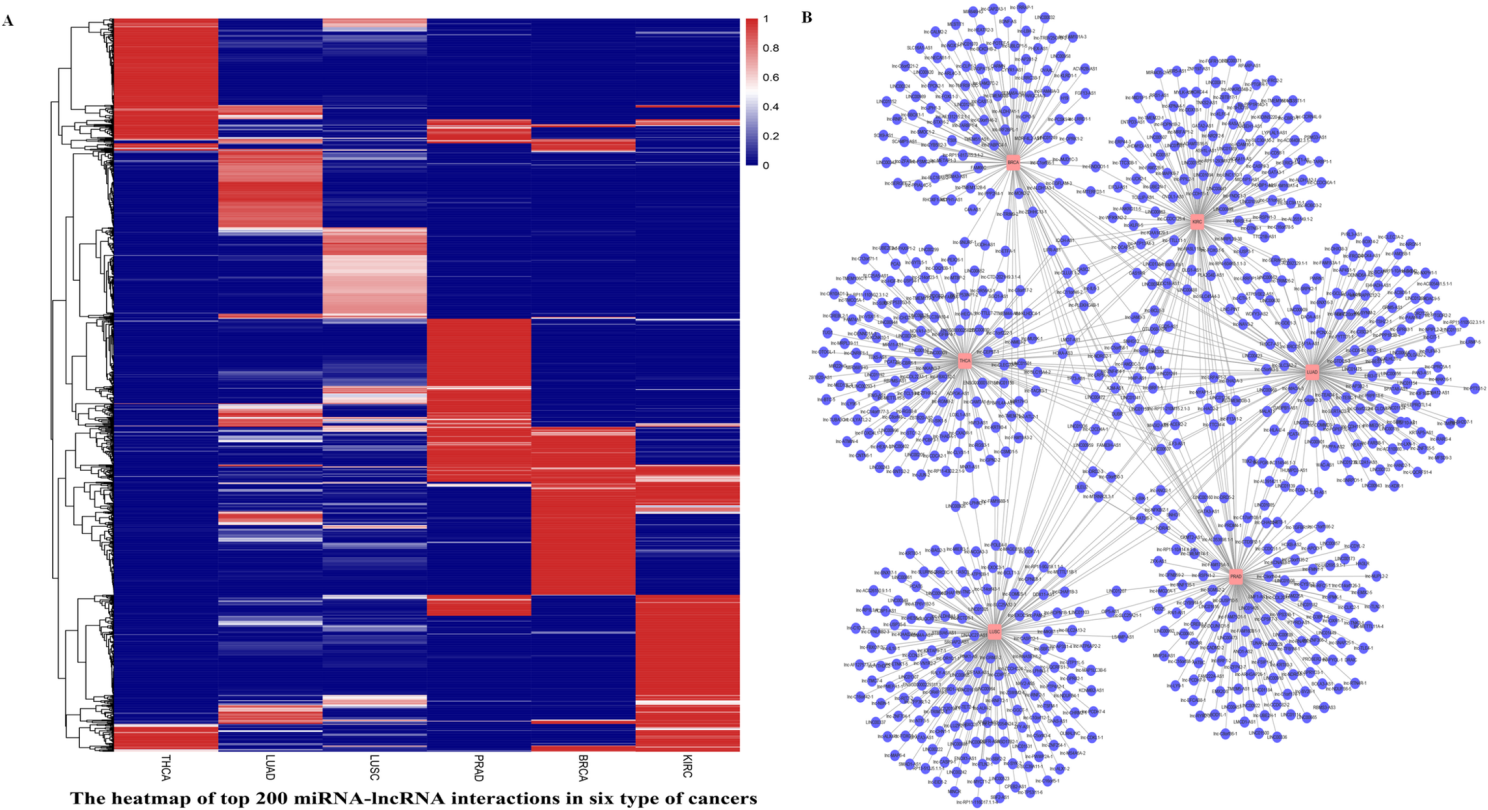
(A) A heat map of the top 200 miRNA-lncRNA interactions in the six types of cancers. Each column represents a cancer, and each row represents the significance score of the corresponding miRNA-lncRNA pair. The redder the color, the greater the significance score of the corresponding pair. (B) The distribution of lncRNA biomarkers involved in the top 200 edge biomarkers in distinct cancers.

To evaluate the distribution of edge biomarkers in different cancers, we chose six types of cancer (BRCA, KIRC, LUAD, LUSC, THCA, and PRAD) to construct cancer-specific miRNA-lncRNA networks. We collected the significance scores of all miRNA-lncRNA pairs in the top 200 miRNA-lncRNA interactions for these six cancers and drew a heat map of the top 200 miRNA-lncRNA interactions for these cancers based on the selected significance scores. As shown in Fig 8A, each type of cancer has a cancer-specific module. It is worth noting that while LUAD and LUSC are both subtypes of lung cancer, we were able to identify subtype-specific modules using our method. In addition, we extracted the top 200 lncRNAs for each of the six types of cancer and drew a network using Cytoscape. It is clear that each cancer has a specific module, the lncRNA DUBR is related to KIRC, LUAD, LUSC and THCA by our prediction, with a degree of 4. Current knowledge of DUBR is minimal, but it is known to interact with mir-210, mir-508, mir-34c and mir-505 in potential edge biomarkers. Many studies have found that these miRNAs play important roles in the progression of cancers [62–66]. For example, mir-210 regulates ISCU by targeting hypoxia-inducible factors 1 and 2 in renal cancer, and mir-505 inversely regulates FZD4 to modulate cancer proliferation and migration in human lung cancer. Moreover, mir-34c is a member of the mir-34 family. Mature mir-34a of the mir-34 family is a part of the p53 tumor suppressor network; thus, dysregulated mir-34 is involved in the development of some cancers [67, 68]. In addition, there were many lncRNAs of degree 1, which we expect to interact specifically with certain miRNAs in subtypes of cancer. For example, H19 is specific for BRCA among the top 200 miRNA-lncRNA pairs; the down-regulation of H19 significantly decreases breast cancer cell clonogenicity and anchorage-independent growth [69–71].

## Discussion

Traditional medicine is dependent on the use of clinical symptoms and signs, combined with sex, age, height, weight, family history, and laboratory and imaging data, to determine the dosage and dosage form of drugs. This is typically a passive approach, wherein treatment or medication is initiated after the symptoms and signs that have already appeared. However, personalized medicine is based on personal genome information, combined with proteome, metabolome and other relevant information affording a view of the internal environment, which is used to tailor a treatment plan for maximum therapeutic effect and minimal side effects. Traditional medicine is not preventative but rather reacts after the disease occurs. However, personalized medicine is best for a certain type of patient who benefits from individual-specific diagnosis and therapy. Personalized medicine can provide a more effective and targeted treatment, and can prevent the occurrence of certain diseases through more accurate diagnosis. Cancer mortality remains high because the cause of cancer is not fully understood. Thus, the development of an effective method for the identification of cancer-related biomarkers is urgently needed [72]. Here, we created an ISMLN for each BRCA sample and a BMLN for each type of cancer by using RNA expression profiles obtained from the TCGA database. We then selected candidate miRNA-lncRNA pairs using a significance score. The potential miRNA-lncRNA pairs were confirmed by feature importance. For BRCA, 111/120 miRNAs predicted as potential miRNA-lncRNA biomarkers were related to BRCA; thus, these miRNA-related edges are likely to be predictive. Through the classification model, we found that these potential biomarkers indeed perform well, as shown below:

1. BMLN can mine non-differentially expressed miRNAs, which play important roles in cancer.
  Although most genes are not differentially expressed, BMLN is capable of identifying non-differentially expressed miRNAs and lncRNAs that play important roles in cancer. This development is pivotal for mining functional genes.
2. BLMNs of different types of cancers hold different significance.
  In this study, we found that the BMLN of six cancers differ significantly by performing a clustering analysis of the top 20 miRNA-lncRNA interactions for the six different types of cancer. The result indicated that the BMLN of each cancer is very specific. This can help us to distinguish the type and subtype of each cancer.
3. The edge biomarkers contain more information than the node biomarkers.
  The classification ability of miRNA-lncRNA edge biomarkers is significantly better than that of node biomarkers (miRNAs and lncRNAs) in the BMLN. This result shows that edge biomarkers contain more biological information.

Overall, the biomarkers selected by us are reliable. The researchers can give priority to study the mechanism of biomarkers selected by our method in the future experiments. This enables researchers to find the biological mechanisms behind a disease and use them directly as a tool for diagnosis and prognosis. In addition, our research will conducive to the development of biopharming and precision medicine. Moreover, researchers can monitor the status of interaction network based on the identified miRNA-lncRNA biomarkers, we can prevent and diagnosis the development for cancer in time, and give the specific treatment for every people.

Our study is limited by several bottlenecks. There are 2588 miRNAs in the miRBase, but only 1046 of these miRNAs are represented in the TCGA database. Moreover, the information describing lncRNAs is relatively limited, the expression profiles of some lncRNAs are unavailable, and the validation of lncRNAs is difficult. Finally, the original miRNA-lncRNA associations are incomplete. All of these factors affect the construction of the ISMLN.

In general, we can construct reliable ISMLNs and BMLNs using our method. Based on the differential miRNA-lncRNA network, we can identify candidate miRNA-lncRNA pairs by significance score. Furthermore, we can select potential miRNA-lncRNA pairs based on their feature importance. These potential miRNA-lncRNA pairs should be prioritized in future efforts at experimental validation. Moreover, these potential miRNA-lncRNA pairs will become therapeutic targets of cancers, and can help us to understand the mechanism of cancers.

## Supporting information

S1 Table The main clinicopathological characteristics of 6 cancer cohorts. (XLSX)

S2 Table The 8508 miRNA-lncRNA pairs of BRCA in the candidate biomarkers. (XLSX)

S3 Table The Activity Score of 240 miRNAs in candidate interactions. (XLSX)

S4 Table The main information of available data resources. (XLSX)
